# Comparative Evaluation of 16S rRNA Target Regions Using PCR High-Resolution Melt Curve Analysis for Differentiation of Bacteria Associated with Bovine Mastitis

**DOI:** 10.3390/microorganisms14091945

**Published:** 2026-09-02

**Authors:** Tewodros Fentahun Jember, Mark Edward Westman, Sameer Dinkar Pant, Seyed Ali Ghorashi

**Affiliations:** 1 School of Agricultural, Environmental and Veterinary Sciences, Faculty of Science and Health, Charles Sturt University, Wagga Wagga, NSW 2678, Australia; tjember@csu.edu.au (T.F.J.); spant@csu.edu.au (S.D.P.); 2 College of Veterinary Medicine and Animal Sciences, University of Gondar, Gondar P.O. Box 196, Ethiopia; 3 Elizabeth Macarthur Agricultural Institute (EMAI), Department of Primary Industries and Regional Development (DPIRD), Menangle, NSW 2568, Australia; mark.westman@dpird.nsw.gov.au; 4 Gulbali Institute, Charles Sturt University, Boorooma Street, Wagga Wagga, NSW 2678, Australia

**Keywords:** high-resolution melt analysis, bovine mastitis, bacterial identification, PCR, diagnostic microbiology, veterinary science

## Abstract

Bovine bacterial mastitis remains a major challenge for the dairy industry, and timely identification of causative bacteria is essential for effective disease management. This study compared six regions of the 16S rRNA gene for PCR high-resolution melt (HRM) curve analysis to identify the most suitable target for species-level differentiation of mastitis-associated bacteria. A total of 547 clinical bovine milk samples from cows with clinical mastitis were cultured, and representative isolates of 18 bacterial species were selected for comparative analysis. Species identity was confirmed using matrix-assisted laser desorption ionisation time-of-flight mass spectrometry (MALDI-TOF MS) prior to molecular analysis. All six primer pair combinations were tested across the 18 species panel, and discriminatory performance was assessed using a genotype confidence percentage (GCP)-based classification model that allowed for objective interpretation without visual inspection. Among the six target regions, the 203 bp amplicon generated by primer pair F1R1, spanning the V1-to-V2 region and part of V3, showed the strongest overall discriminatory performance with no cross-classification observed. Sequencing of F1R1 amplicons confirmed species identity and showed concordance with HRM-based classification. Phylogenetic analysis of sequences from this region showed clustering broadly consistent with established taxonomic relationships. Proof-of-concept testing showed that the workflow could also be applied to DNA extracted directly from selected milk samples without prior bacterial isolation. Overall, this approach provides a rapid, cost-effective, and high-throughput method for preliminary discrimination of mastitis-associated bacteria.

## 1. Introduction

Bovine mastitis is a multifactorial disease of major economic and animal welfare importance in the dairy industry worldwide [1,2]. It is primarily caused by a diverse range of bacterial pathogens, including Gram-positive, Gram-negative, and atypical organisms, which differ in pathogenicity, treatment response, and epidemiological significance. Rapid and accurate identification of the causative agent is therefore essential for effective clinical management, antimicrobial stewardship, and herd-level disease control [3]. However, the diversity of bacteria associated with mastitis continues to challenge routine diagnostic workflows, particularly when timely results are required. Moreover, species-level identification is clinically important because mastitis pathogens differ in their epidemiology, antimicrobial susceptibility, virulence, and treatment response, enabling targeted therapy, improved herd management, and more effective mastitis control and antimicrobial stewardship [4,5]. Furthermore, contagious pathogens such as *Staphylococcus aureus* often require different control strategies from environmental pathogens such as *Escherichia coli* and *Streptococcus uberis*. Accurate species identification enables veterinarians to select appropriate antimicrobial therapy, avoid unnecessary or ineffective treatments, predict disease outcome, and implement pathogen-specific control measures, including improvements in milking hygiene, environmental management, and culling decisions [6,7,8].

Conventional bacterial culture remains the reference method for mastitis diagnosis, but is time-consuming and may fail to detect slow-growing, fastidious, or non-viable organisms [9]. Molecular approaches, particularly PCR-based methods, have been increasingly used for bacterial identification because of their high sensitivity and specificity [10,11]. Sequencing of the 16S rRNA gene is widely used as a reference method for bacterial taxonomic identification and phylogenetic placement, enabling robust assignment through comparison with curated reference databases, although species-level resolution is not always achievable [12,13]. However, despite its accuracy, sequencing-based identification is less practical for routine diagnostic use because of cost, turnaround time, post-PCR processing requirements, and the need for specialised expertise, which can restrict its use in high-throughput diagnostic laboratories [14].

PCR coupled with high-resolution melt (PCR-HRM) curve analysis has emerged as a promising alternative for rapid bacterial differentiation [15]. HRM distinguishes organisms based on sequence-dependent differences in melting behaviour within amplified DNA fragments, generating characteristic conventional and normalised melt curve profiles [16,17]. When applied to conserved genes containing variable regions, such as the 16S rRNA gene, HRM enables closed-tube, real-time discrimination of bacterial species without the need for sequencing [18,19]. The technique offers several practical advantages, including reduced turnaround time, lower cost, minimal contamination risk, and compatibility with high-throughput workflows, making it potentially suitable for some routine diagnostic applications [15,20]. The application of 16S rRNA-based PCR-HRM for mastitis pathogen identification was first demonstrated by Ajitkumar, et al. [21], who reported the feasibility of differentiating nine mastitis-associated bacterial pathogens using HRM analysis of a single 16S rRNA fragment. While this study provided an important proof of concept, it was based on a limited number of isolates, and not all isolates were derived from clinical bovine milk samples. In addition, discrimination was assessed using a single target region, and curve interpretation relied primarily on visual assessment of melt profiles rather than an objective, mathematically defined classification approach, which may limit scalability and reproducibility in routine diagnostic settings [21,22]. Subsequent studies have demonstrated that the discriminatory power of HRM is strongly influenced by the choice of target region, amplicon length, and underlying sequence variability within the amplified fragment [17,23,24]. Species-level discrimination using the 16S rRNA gene can be limited by high sequence similarity among closely related species, multiple gene copies with intragenomic heterogeneity, intraspecific variation, and overlapping HRM melting profiles, highlighting the importance of selecting appropriate target regions [25,26].

Closely related species may exhibit overlapping melt profiles when a single region is used, whereas targeting alternative or multiple regions can improve resolution. Despite this, to our knowledge, a comprehensive evaluation of multiple 16S rRNA regions for HRM-based differentiation of mastitis-associated pathogens using a large collection of clinically confirmed bovine milk isolates has not been reported.

Therefore, the aim of this study was to compare six different regions of the 16S rRNA gene for their suitability in PCR-HRM-based species-level differentiation of bacteria associated with bovine mastitis. Discriminatory performance was assessed using a genotype confidence percentage (GCP)-based mathematical model for objective curve classification. Agreement between HRM-based identification and DNA sequencing was also evaluated.

We hypothesised that different 16S rRNA target regions have variable discriminatory power and that selecting an optimal region can improve species-level differentiation of bovine mastitis-associated bacteria using PCR-HRM analysis.

## 2. Materials and Methods

### 2.1. Clinical Samples and Bacterial Isolates

A total of 547 clinical bovine milk samples were received by the Elizabeth Macarthur Agricultural Institute (EMAI), Department of Primary Industries and Regional Development, Menangle, New South Wales, Australia, between 2023 and 2025 from cows with suspected clinical mastitis. Individual animal-level identifiers were not consistently available for these diagnostic submissions, so it was not possible to confirm the exact number of individual cows represented by the 547 samples or to rule out quarter-level duplicate submissions from the same animal. All samples were processed as part of routine diagnostic investigations. Milk samples were cultured using standard bacteriological methods, and bacterial isolates were obtained from culture-positive samples. Briefly, 10 µL of each milk sample was inoculated onto a sheep blood agar (SBA) plate and 10 µL onto a MacConkey agar plate; both plates were incubated aerobically at 37 °C for 48 h. Bacterial growth was recorded at 48 h as sparse (1+), moderate (2+), or profuse (3+), and classified as pure growth (a single colony type), mixed growth (two or more colony types), or predominant growth. Plates showing three or more different colony types with no single predominant organism were considered contaminated and reported as no significant growth. If there was no growth on either plate after 24 h, the milk sample was inoculated again onto fresh plates using the same procedure, having been incubated at 37 °C since receipt (enriched culture). Colonies selected for identification were sub-cultured onto a fresh SBA plate and incubated aerobically with 5% CO_2_ for 24 to 48 h to obtain pure growth for MALDI-TOF MS. These clinical samples served as the source population from which representative isolates were selected for method development and comparison. From the culture-positive samples, a representative isolate panel comprising 18 mastitis-associated bacterial species was assembled for comparative PCR-HRM evaluation (Table S1). Of the 547 milk samples received, samples were processed by bacterial culture, and culture-positive isolates were identified to the species level. Representative isolates from the identified mastitis-associated bacterial species were selected based on availability and confirmed identification. These isolates were subsequently used for PCR-HRM analysis to evaluate the discriminatory performance of different 16S rRNA target regions. One representative isolate was tested per species across all six primer pairs (Table S1).

Most isolates in this panel were recovered from the bovine milk samples described above, while two were derived from caprine milk samples. These were *Streptococcus agalactiae* and *Providencia stuartii* (identified as caprine-derived in Table S1). These were included because they represent important mastitis-associated species relevant to species-level discrimination. The *Streptococcus agalactiae* isolate (IFM 3200) was additionally sourced from the IFM culture collection rather than directly from EMAI diagnostic submissions. The host of origin for all isolates is annotated in Supplementary Table S1. Pure colonies were sub-cultured as required to ensure isolate purity prior to DNA extraction.

### 2.2. Bacterial Identification and Reference Confirmation

Species identity of the selected isolates was confirmed at EMAI using MALDI TOF MS (Bruker MALDI Biotyper System 4.0; Bruker Daltonics, Billerica, MA, USA) with established reference databases. A single colony was applied to a Bruker MSP 96 polished steel target plate (Cat. No. 8280800, Bruker, Melbourne, Australia) using a sterile applicator, overlaid with 1 µL of 70% formic acid (Sigma-Aldrich, St. Louis, MO, USA, F0507) and allowed to dry, followed by 1 µL of Bruker HCCA matrix. Species identification was performed using the MBT Compass software (Version 13) with Flex Control, referenced against the BDAL library. *Staphylococcus aureus* ATCC 9144 and *Escherichia coli* ATCC 25922 (ThermoFisher Scientific, Waltham, MA, USA) were included as positive controls on every run, and a Bruker Bacterial Test Standard was run weekly. Only identifications with a score greater than 2.0 were accepted as valid species-level results; no ambiguous results requiring further resolution were obtained in this data set. MALDI-TOF MS served as the reference method for bacterial classification prior to molecular analysis. Isolates were selected from the most frequently cultured bovine mastitis pathogens identified in milk samples submitted to the EMAI laboratory from dairy farms in NSW. Selection was based on clinical relevance, taxonomic diversity, the availability of pure isolates, and high-confidence MALDI-TOF MS identification. Hence, only species-confirmed isolates were included in the PCR-HRM evaluation, after which bacterial cultures and corresponding milk samples were transferred to the research laboratories (Charles Sturt University, Wagga Wagga, NSW, Australia) for DNA extraction, PCR-HRM analysis, and sequencing-based validation. All samples and cultures were transported under refrigerated conditions and were either processed immediately or stored at 4 °C until further analysis.

### 2.3. DNA Extraction from Bacterial Isolates and Milk Samples

Genomic DNA was extracted from bacterial isolates using the Wizard Genomic DNA Purification Kit (Promega Corporation, Madison, WI, USA), following the manufacturer’s instructions. Briefly, DNA was extracted from pure bacterial colonies obtained from culture-positive milk samples. The concentration and purity of extracted DNA were assessed using a spectrophotometer, and DNA concentrations were standardised to 5 ng/µL prior to downstream molecular analysis.

In addition to the 18 pure bacterial isolates, DNA was also extracted from four raw milk samples collected prior to culture that had been confirmed as culture-positive and species identified by MALDI-TOF MS. These samples included *Escherichia coli*, *Streptococcus uberis*, *Staphylococcus aureus*, and *Staphylococcus chromogenes*, and were selected to represent common mastitis-associated pathogens. DNA extracted directly from milk was used as a proof-of-concept assessment to evaluate the technical feasibility of applying the PCR-HRM assay directly to clinical milk samples, rather than as a comprehensive validation of assay performance. All extracted DNA samples were stored at −20 °C until further use.

### 2.4. Primer Design and Target Regions of the 16S rRNA Gene

The 16S rRNA gene was selected as the molecular target for bacterial differentiation due to its conserved structure interspersed with variable regions that enable species-level discrimination. Primer design was performed in Geneious version 10.2.6 (Biomatters). Representative 16S rRNA gene sequences from 14 of the 18 target bacterial species were retrieved from GenBank for alignment (*Escherichia coli*, NR_024570.1; *Staphylococcus aureus*, NR_118997.2; *Klebsiella pneumoniae*, NR_036794.1; *Streptococcus uberis*, AB023573.1; *Streptococcus dysgalactiae*, AB002484.1; *Corynebacterium bovis*, NR_037042.1; *Mycoplasma bovis*, NR_102850.2; *Streptococcus agalactiae*, NR_040821.1; *Serratia marcescens*, NR_036886.1; *Pasteurella multocida*, NR_041809.1; *Pseudomonas aeruginosa*, MK073895.1; *Staphylococcus hyicus*, NR_036905.1; *Providencia stuartii*, NR_024848.1; and *Pantoea agglomerans*, NR_041978.1), together with four additional non-target reference sequences (*Enterococcus faecalis*, NR_040789.1; *Lactococcus lactis*, NR_040955.1; *Bacillus cereus*, NR_074540.1; and *Trueperella pecoris*, NR_181460.1) selected to broaden the phylogenetic diversity of the alignment. *Bacillus cereus* was additionally tested by PCR-HRM across all six primer pairs as a non-target specificity control, given that its sequence had already been included in the design alignment. Reference sequences were not available for the remaining four panel species (*Staphylococcus chromogenes*, *Mannheimia haemolytica*, *Staphylococcus simulans*, and *Trueperella pyogenes*) at the design stage; these species were not directly represented in the alignment, but successful amplification with all six primer pairs was subsequently confirmed empirically across the full 18-species panel, including these four species (Section 3.1). Sequences were aligned to identify conserved regions flanking the target hypervariable regions, using Geneious default primer design parameters together with visual inspection of the alignment to confirm conserved primer-binding sites. Hairpin formation, self-dimer, and cross-dimer potential were assessed for all primers using IDT OligoAnalyzer 3.1, (Integrated DNA Technologies), and all primers fell within acceptable thresholds. In silico specificity was confirmed by blast 2.17.0 search of each primer sequence against the NCBI nucleotide database restricted to the 18 target species, with no significant off-target matches identified. Because the resulting primers target universally conserved flanking regions, their binding sites occur at homologous positions across the 18-species panel. Primer coordinates are therefore reported relative to the *Escherichia coli* 16S rRNA reference sequence (NR_024570.1), following the widely used *E. coli* numbering convention [27], F1, 326–343; R1, 509–527; F2, 509–527; R2, 774–797; F3, 774–797; R3, 1089–1104.

Primer binding sites were selected within conserved regions to ensure consistent amplification across species, while the intervening variable regions were targeted to maximise sequence-dependent differences detectable by PCR-HRM analysis. Based on this analysis, three primer sets were designed and subsequently combined in different forward and reverse configurations to generate six distinct amplicons targeting different regions of the 16S rRNA gene for comparative PCR-HRM analysis (Table 1).

### 2.5. PCR Amplification Conditions

PCR amplification conditions were initially evaluated using gradient PCR for each of the six primer pairs to confirm suitable annealing temperatures, after which a uniform amplification protocol was applied for all reactions. Each reaction contained 5 µL of 5× PCR buffer, 1.5 µL of MgCl_2_ (25 mM), 2 µL of dNTP mix (2 mM), 0.3 µL of Taq DNA polymerase (5 U/µL) (GoTaq^®^ DNA Polymerase; Promega Corporation, Madison, WI, USA), 1 µL each of the forward and reverse primers (2 µM), and 2 µL of a working SYTO 9 green fluorescent nucleic acid stain solution (Invitrogen, Thermo Fisher Scientific, Carlsbad, CA, USA), prepared by diluting the manufacturer’s 5 mM stock in DMSO 1:50 in nuclease-free water to 100 µM and adding 2 µL per 25 µL reaction, for a final concentration of 8 µM, and 2 µL of template DNA. Nuclease-free water was added to achieve the final reaction volume of 25 µL. Negative controls were included in each run to monitor contamination. For PCR-HRM analysis of DNA extracted from bacterial cultures, the negative control was a no-template control containing nuclease-free water.

For PCR-HRM analysis of DNA extracted directly from clinical milk samples, DNA extracted from pasteurised milk was used as a milk-matrix control rather than a true negative control, since pasteurisation does not eliminate residual bacterial DNA. PCR amplification was carried out using an initial denaturation step at 95 °C for 3 min, followed by 36 cycles of denaturation at 95 °C for 30 s, annealing at 55 °C for 30 s, and extension at 72 °C for 30 s, with a final extension step at 72 °C for 5 min. PCR amplicons were analysed by PCR-HRM analysis, and representative products were visualised by agarose gel electrophoresis to confirm expected amplicon sizes.

### 2.6. High-Resolution Melt Curve Analysis

High-resolution melt curve analysis was performed immediately following PCR amplification using a Rotor-Gene 6000 real-time PCR system (Qiagen, Clayton, VIC, Australia). HRM analysis was conducted for all primer pair combinations to evaluate their ability to differentiate bacterial species based on the melting behaviour of amplified 16S rRNA gene fragments. PCR products were subjected to HRM analysis using three different temperature ramping rates of 0.1, 0.2, and 0.3 °C s^−1^ to assess melting behaviour under varying conditions. Fluorescence data were continuously acquired during melting to generate conventional and normalised melt curves using Rotor-Gene version 1.7.27 (Qiagen, Clayton, VIC, Australia). Normalisation was performed using a pre-melt temperature range of 60–95 °C for all amplicons; primer-pair-specific post-melt ranges were F1R1, 83–92 °C; F2R2, 82–92 °C; F3R3, 82–94 °C; F1R3, 84–95 °C; F1R2, 83–93 °C; and F2R3, 83–95 °C.

Melting temperature values were derived from the normalised melt curves, and differences in curve shape and melting profiles were used to assess discrimination among bacterial species. HRM analysis was performed in triplicate for each isolate, and analyses were repeated across independent runs to assess reproducibility. All HRM analyses were performed using identical instrument settings and reaction conditions to ensure consistency across primer sets and bacterial species.

### 2.7. Automated Curve Classification and Genotype Confidence Percentage Analysis

PCR-HRM analysis was performed across multiple independent experimental runs conducted at different times to assess reproducibility. For each primer pair, one bacterial species was designated in turn as the reference genotype, and GCP values were obtained for all 18 species included in the study. This process was repeated so that each bacterial species served as the reference genotype for analysis. GCP values obtained across repeated runs were used to calculate the mean and standard deviation for each reference species. GCP was calculated by comparing each test HRM profile with reference profiles and expressing the degree of similarity as a percentage. Each isolate was assigned to the species showing the highest GCP value, provided it met the predefined classification threshold.

To establish a conservative and reproducible classification threshold, a species-specific GCP cut-off value was defined for each bacterial species. For each reference species, the cut-off value was calculated from the mean GCP obtained when that species was matched against itself across repeated HRM analyses, minus three times the corresponding standard deviation. This approach was used to account for inter-run variation when defining the cut-off threshold and to reduce the likelihood of misclassification.

The species-specific cut-off values were subsequently applied during HRM analysis to evaluate the discriminatory power of each primer pair and to assess the ability of the assay to reliably differentiate a given bacterial species from the remaining 18 species included in the study. This strategy allowed HRM profile discrimination to be performed using statistically defined thresholds rather than subjective visual interpretation.

Samples were assigned to a bacterial species genotype when the genotype confidence percentage value was equal to or greater than the corresponding species-specific cut-off value and represented the highest GCP among all candidate species. When the GCP value was lower than the corresponding cut-off, the isolate was classified as “variation” according to the GCP-based classification approach. The full set of species-specific GCP cut-off values for each primer pair is provided in Supplementary Table S2.

### 2.8. DNA Sequencing and Nucleotide Sequence Analysis

Following the comparative HRM and GCP evaluation, PCR amplicons generated using the F1R1 primer pair were submitted for bidirectional DNA sequencing at the Australian Genome Research Facility, Brisbane, Australia. The F1R1 primer pair was selected for sequencing because it demonstrated the highest discriminatory performance among the six combinations tested. Sequencing was used to confirm the genetic basis of HRM-based classification. Raw sequence data were quality-checked and assembled to generate consensus sequences for each bacterial species. Sequence identity was assessed by comparison with reference sequences available in public databases using BLAST analysis. Nucleotide sequences generated in this study were submitted to GenBank, and accession numbers were assigned for each bacterial species analysed. Sequence alignment was conducted using CLUSTAL W [28] with a gap open penalty of 10 and a gap extension penalty of 1, and aligned sequences were further examined using BioEdit Sequence Alignment Editor version 6.0.9.0. The aligned sequences were used to construct a maximum likelihood phylogenetic tree in Molecular Evolutionary Genetics Analysis (MEGA software, version 10) with node support assessed using 1000 bootstrap replicates, to assess genetic relatedness among the bacterial species included in the study.

## 3. Results

### 3.1. PCR Amplification and HRM-Based Discrimination Across 16S rRNA Regions

**Figure 1:**
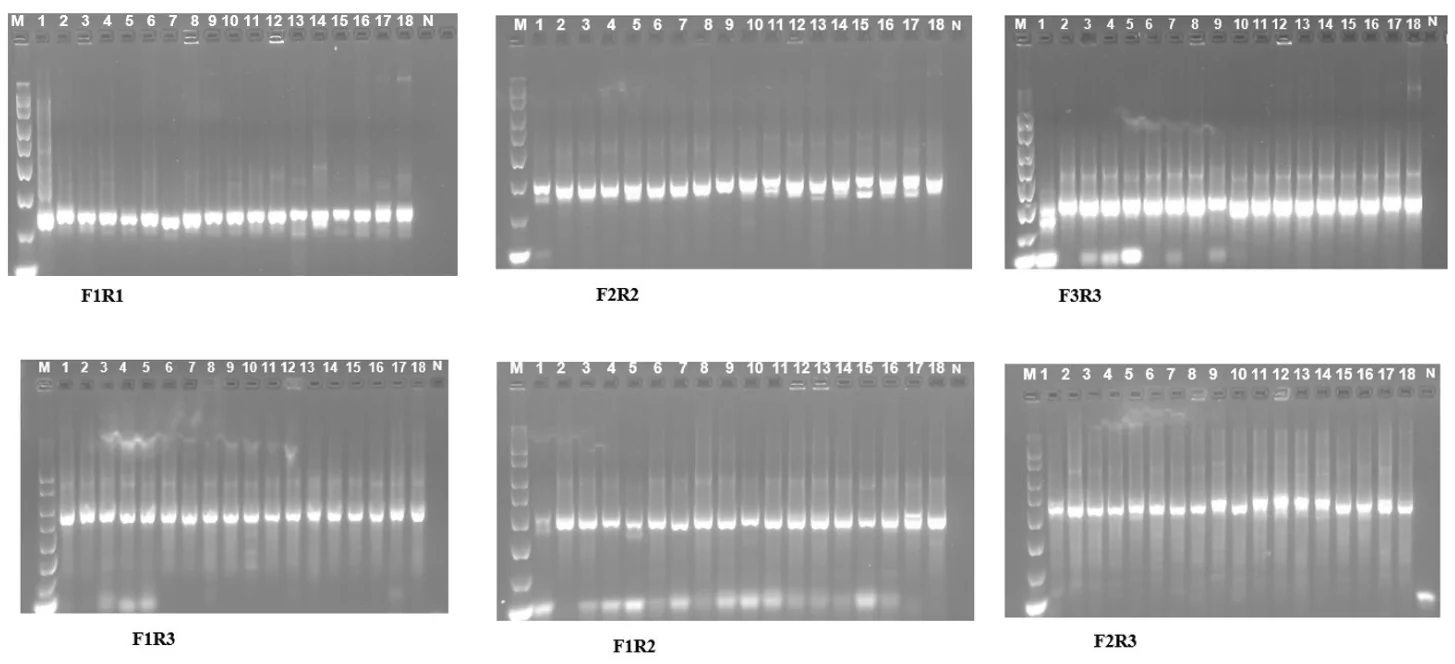
Agarose gel electrophoresis results for six different 16S rRNA target regions. Gels were prepared using 1.0 to 2.0% agarose (LE Analytical Agarose, Promega Corporation, Madison, WI, USA) and 2% GelRed nucleic acid stain (10,000× in water; Fisher Biotec, Wembley, WA, Australia). The expected amplicon sizes were 203, 293, 323, 779, 473, and 599 bp for F1R1, F2R2, F3R3, F1R3, F1R2, and F2R3, respectively. M indicates the DNA ladder in lane 1, and N indicates the negative control. Samples 1 to 18 correspond to *Escherichia coli* (1), *Streptococcus uberis* (2), *Staphylococcus aureus* (3), *Streptococcus agalactiae* (4), *Corynebacterium bovis* (5), *Serratia marcescens* (6), *Trueperella pyogenes* (7), *Staphylococcus chromogenes* (8), *Mannheimia haemolytica* (9), *Mycoplasma bovis* (10), *Staphylococcus simulans* (11), *Staphylococcus hyicus* (12), *Providencia stuartii* (13), *Pantoea agglomerans* (14), *Streptococcus dysgalactiae* (15), *Klebsiella pneumoniae* (16), *Pasteurella multocida* (17), and *Pseudomonas aeruginosa* (18).

PCR amplification was successfully achieved for all 18 bacterial species using each of the six primer pair combinations targeting different regions of the 16S rRNA gene. Agarose gel electrophoresis confirmed amplification of the six targeted regions, with each primer pair producing bands of the expected size (Figure 1). Amplification was reproducible across technical replicates and independent runs.

**Figure 2:**
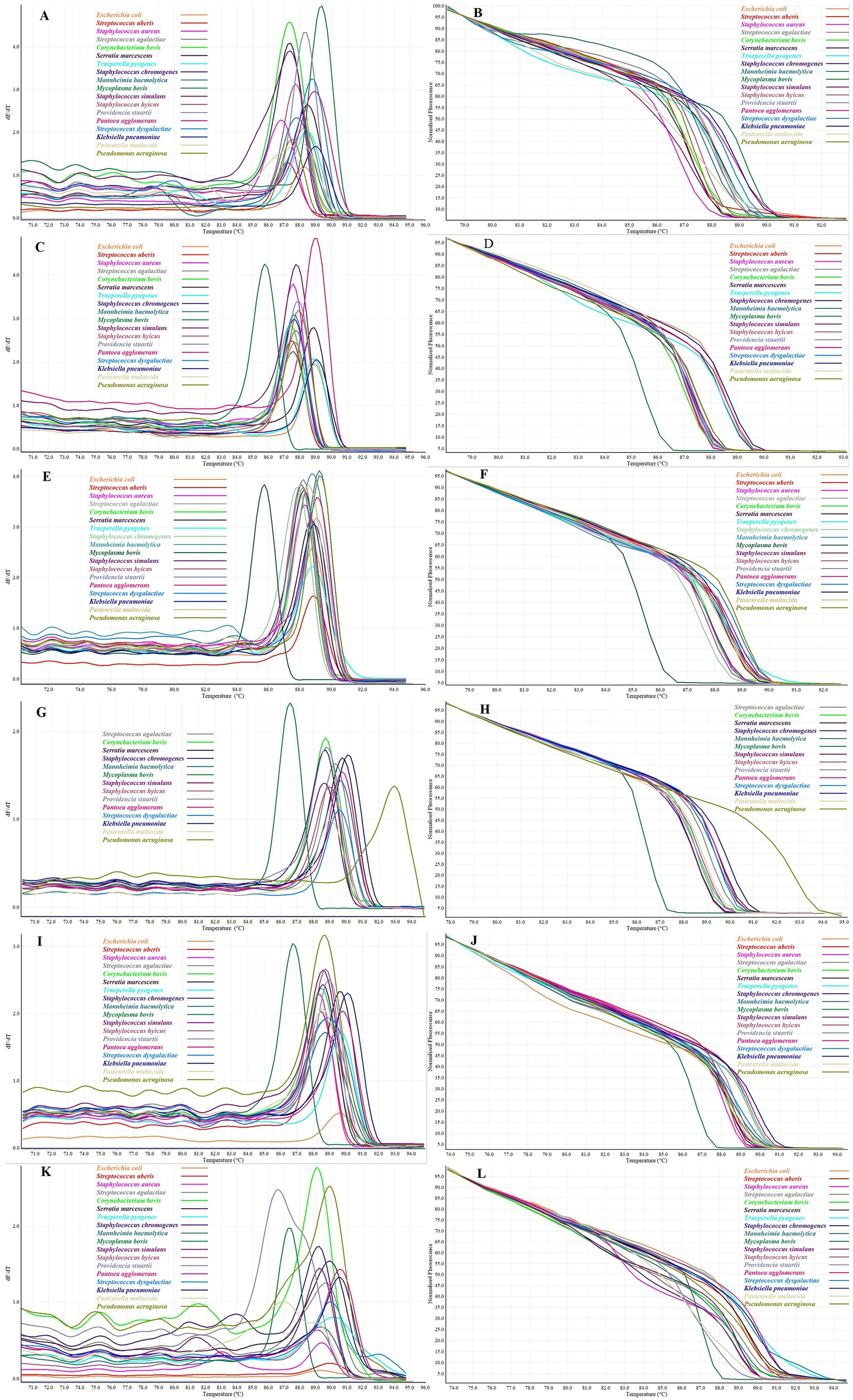
Conventional (**A**,**C**,**E**,**G**,**I**,**K**) and normalised (**B**,**D**,**F**,**H**,**J**,**L**) HRM curve profiles generated using six 16S rRNA primer pair combinations for 18 mastitis-associated bacterial species. Panels (**A**,**B**) correspond to primer pair F1R1, panels (**C**,**D**) to F2R2, panels (**E**,**F**) to F3R3, panels (**G**,**H**) to F1R3, panels (**I**,**J**) to F1R2, and panels (**K**,**L**) to F2R3.

High-resolution melt curve analysis generated clear and consistent conventional and normalised melt profiles for all primer sets. Comparison of melt curves obtained at different temperature ramp rates indicated that a ramping rate of 0.3 °C s^−1^ resulted in clearer separation of melt profiles for the majority of bacterial species across all six primer sets. Across HRM analyses, most bacterial species produced a single, well-defined melting peak, indicating uniform melting behaviour of the amplified 16S rRNA gene fragments (Figure 2).

Distinct species-specific differences in melting behaviour were observed across the six primer regions, reflected by variation in melting temperature values and curve shapes. Melting temperature values for all bacterial species and primer pairs are summarised in Table 2. Among the six primer pair combinations, mean melting temperature ranges varied depending on the targeted region of the 16S rRNA gene. *Mycoplasma bovis* consistently exhibited lower melting temperatures across most primer regions, whereas higher melting temperatures were observed for species such as *Klebsiella pneumoniae*, *Pantoea agglomerans*, and *Pseudomonas aeruginosa* for specific primer pairs.

Mean melting temperature ranges varied across the six primer pair combinations, with F3R3 showing the broadest range and F2R2 the narrowest (Table 3). Visual comparison of the conventional and normalised HRM curves showed clear differences in discriminatory performance among the six primer pair combinations. Primer pair F1R1 produced the clearest visual separation among the bacterial species, with more distinct curve profiles and less clustering than the other primer sets (Figure 2A,B). Primer pair F2R3 also showed comparatively strong discriminatory performance, although some overlap between species remained (Figure 2K,L). In contrast, primer pairs F2R2 and F3R3 tended to separate the bacterial panel into broader clusters rather than clearly distinct species (Figure 2C–F), while F1R3 showed intermediate separation and F1R2 displayed comparatively weak visual discrimination (Figure 2G–J). Across these analyses, *Mycoplasma bovis* consistently produced a distinctly lower melting peak than most other species for all primer sets except F1R1, where this separation was less marked.

A concise summary of visual discrimination, GCP classification performance, and overall assay performance across the six primer pair combinations is provided in Table 3. Among the six primer pair combinations, F1R1 showed the strongest overall classification performance, while F2R3 ranked second and also showed strong discrimination, although it was not entirely free from cross-classification. These differences in melting behaviour and profile separation across primer regions were further examined using GCP analysis to enable objective species classification.

### 3.2. Genotype Confidence Percentage Analysis and Species Classification

GCP analysis was used to quantitatively assess HRM-based species discrimination and to enable objective classification across the six 16S rRNA primer pair combinations. For each primer pair, GCP values were calculated for all bacterial species using each species in turn as the reference genotype. Species-specific GCP cut-off values, derived from repeated HRM analyses, as described in the Section 2, were applied to determine correct species assignment. These cut-off values varied across species and primer pairs because they were derived from the mean GCP and corresponding standard deviation across repeated HRM runs for each reference genotype, reflecting differences in run-to-run variability and reference-specific profile consistency. The full updated GCP data and the corresponding species-specific cut-off values for all six primer pairs are provided in Supplementary Table S2.

Using primer pair F1R1 as a representative example, GCP analysis demonstrated clear species-level discrimination across the bacterial panel. When each species was used as the reference genotype, the corresponding isolate consistently produced high mean GCP values that exceeded the species-specific cut-off threshold, indicating correct self-classification. In contrast, GCP values obtained for non-corresponding species remained below the cut-off values, resulting in classification as variation rather than misassignment to the reference genotype.

This pattern was consistent across repeated runs, indicating stable inter-run performance for the F1R1 primer pair. Overall, F1R1 showed the most robust discriminatory performance, with reliable separation between target and non-target species and no cross-classification observed when species-specific cut-off values were applied.

**Figure 3:**
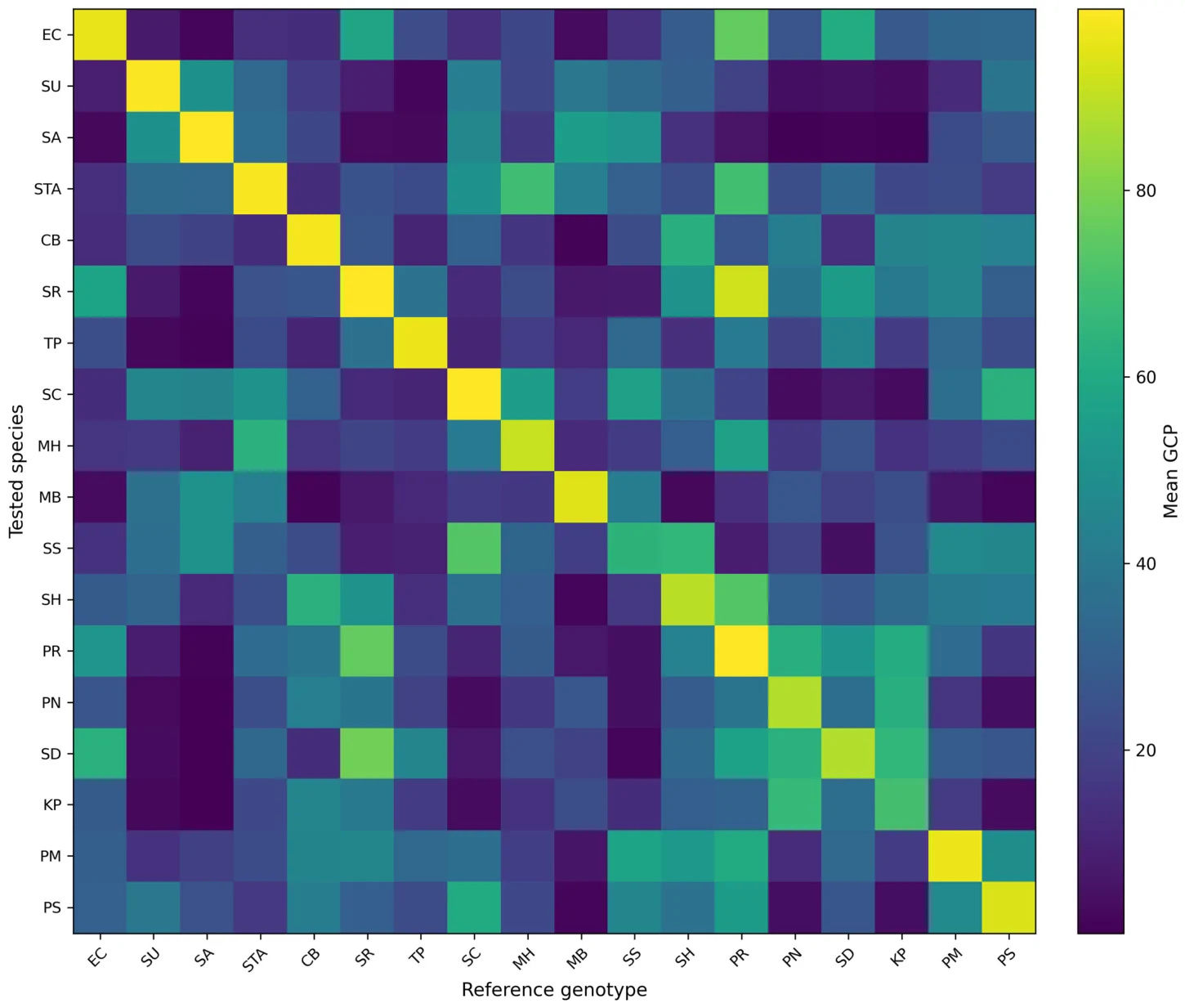
Heatmap of mean genotype confidence percentage values across 18 bacterial species. Mean GCP values are shown for each tested sample against each reference genotype. Higher values indicate stronger similarity to the reference genotype, with the diagonal representing self-match comparisons. Elevated off-diagonal values indicate increased similarity between non-target and reference species. Abbreviations: EC, *Escherichia coli*; SU, *Streptococcus uberis*; SA, *Staphylococcus aureus*; STA, *Streptococcus agalactiae*; CB, *Corynebacterium bovis*; SR, *Serratia marcescens*; TP, *Trueperella pyogenes*; SC, *Staphylococcus chromogenes*; MH, *Mannheimia haemolytica*; MB, *Mycoplasma bovis*; SS, *Staphylococcus simulans*; SH, *Staphylococcus hyicus*; PR, *Providencia stuartii*; PN, *Pantoea agglomerans*; SD, *Streptococcus dysgalactiae*; KP, *Klebsiella pneumoniae*; PM, *Pasteurella multocida*; and PS, *Pseudomonas aeruginosa*.

In contrast, the remaining primer pairs showed varying degrees of cross-classification. F1R3 and F1R2 showed limited cross-classification, whereas F2R2 and F3R3 showed broader overlap across multiple reference genotypes. Primer pair F2R3 also performed well but was not entirely free from cross-classification and therefore showed lower specificity than F1R1 under the applied cut-off-based classification model. To visualise the overall distribution of mean GCP values across the bacterial panel, a heatmap was generated using all tested samples and reference genotypes (Figure 3). The heatmap showed a strong diagonal pattern, with the highest GCP values occurring when each species was compared against its corresponding reference genotype. Off-diagonal values were generally lower, although several reference genotypes showed elevated similarity with selected non-target species, consistent with the cross-classification patterns identified in the cut-off-based analysis.

A supplementary discrimination gap analysis, calculated as the difference between the self-match GCP and the highest non-target GCP for each reference genotype, showed a similar overall pattern of relative discriminatory strength among the reference species (Figure S1).

Taken together, these results demonstrate that GCP analysis provided an objective basis for comparing the discriminatory performance of the six primer pair combinations without reliance on visual curve interpretation. Among the primer sets evaluated, F1R1 showed the most robust classification across the bacterial panel, whereas F1R3 and F1R2 showed limited cross-classification, and F2R2, F3R3, and F2R3 showed broader or selected cross-assignment for some species. GCP values and species-specific cut-off thresholds were consistent across repeated HRM runs, indicating stable inter-run performance and reproducible classification behaviour. *Bacillus cereus* did not cross-classify as any of the 18 target species under any primer pair (Supplementary Table S2), consistent with the assay’s specificity. Based on these comparative outcomes, F1R1 was selected for downstream sequence analysis.

### 3.3. Sequence Confirmation and Phylogenetic Analysis

Sequencing was performed on PCR amplicons generated using the F1R1 primer pair. High-quality sequences were obtained, and all chromatograms showed specific amplification without mixed peaks. BLAST analysis showed that the top matches corresponded to the expected species based on HRM and GCP classification. GenBank accession numbers for the sequenced isolates are provided in Table 2.

**Figure 4:**
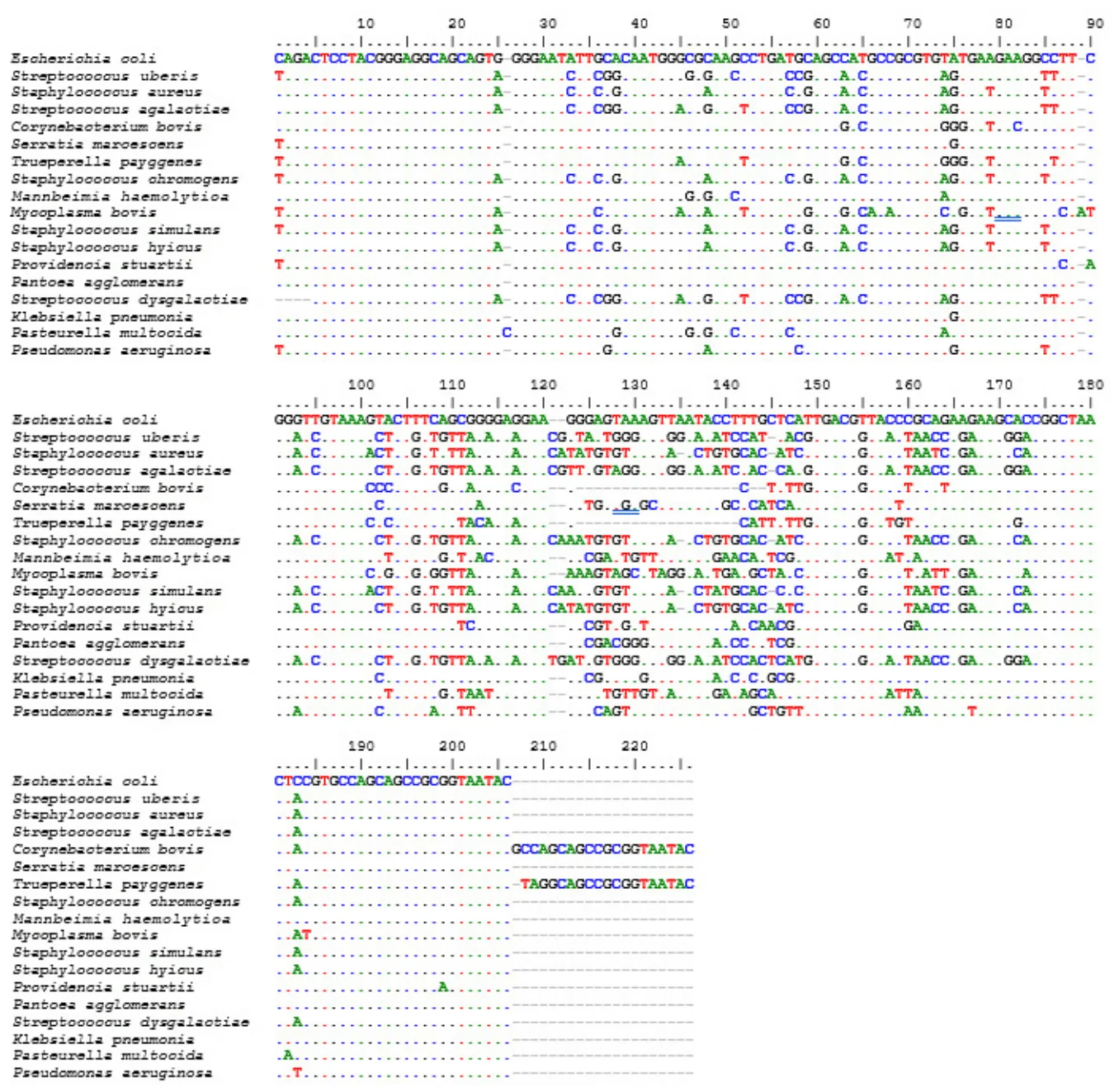
Multiple sequence alignment of the F1R1 amplicon derived from the V1–V2 region and part of the V3 region of the 16S rRNA gene for 18 bacterial species associated with bovine mastitis. Conserved nucleotides are indicated by dots, and gaps are represented by dashes. Nucleotide substitutions are colour-coded by base identity: adenine (green), thymine (red), guanine (black), and cytosine (blue).

Multiple sequence alignment of the F1R1 amplicon sequences showed nucleotide variation across the analysed bacterial species within the targeted V1–V2 region and part of the V3 region of the 16S rRNA gene (Figure 4). Conserved positions were observed across several taxa, while species-specific nucleotide differences were also present within the alignment. These aligned sequences were subsequently used for phylogenetic analysis.

**Figure 5:**
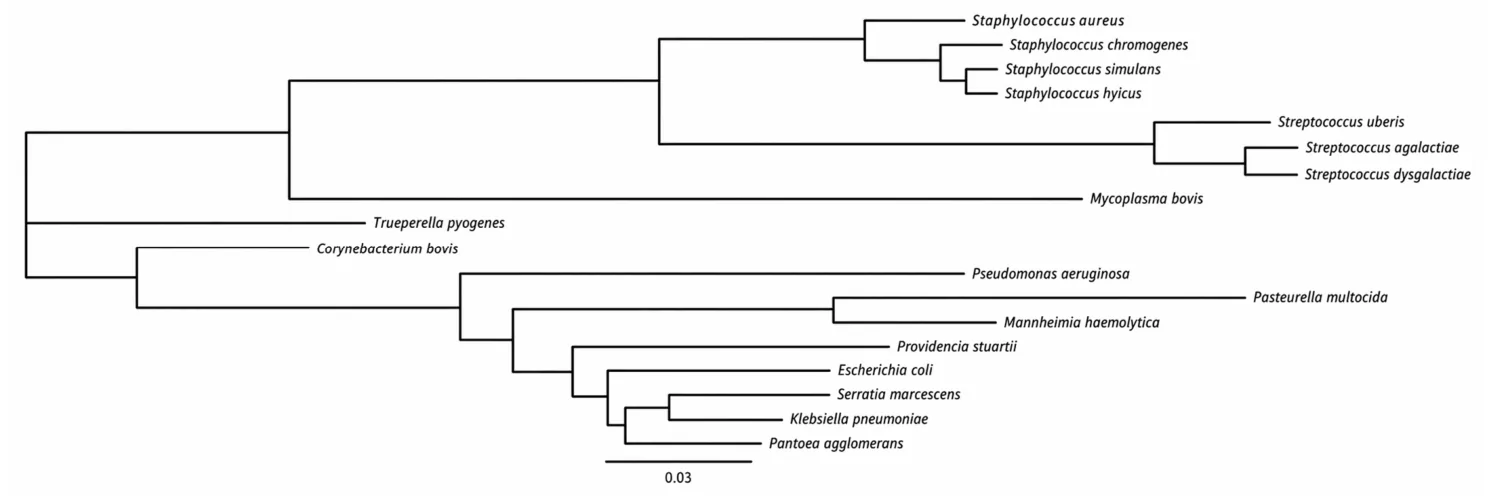
Phylogenetic tree constructed from aligned amplicon sequences generated with the F1R1 primer pair, corresponding to the V1–V2 region and part of the V3 region of the 16S rRNA gene, for 18 bacterial species associated with bovine mastitis. Branch lengths represent sequence divergence, and the scale bar indicates the number of nucleotide substitutions per site. The tree was constructed using the maximum likelihood method in the MEGA software, version 10 with node support assessed using 1000 bootstrap replicates.

A phylogenetic tree constructed from the aligned F1R1 sequences showed clustering of the sequenced isolates according to sequence relatedness (Figure 5). The overall clustering pattern was broadly consistent with established taxonomic relationships among the analysed bacteria. In addition to genus- and family-level grouping, the tree showed broad separation between many taxa traditionally classified as Gram-positive and Gram-negative. This pattern was interpreted as phylogenetic clustering based on partial 16S rRNA sequence relatedness rather than as direct phenotypic classification. Isolates classified as the same species by HRM and GCP analysis clustered within the same clade, indicating that the sequence-based analyses were consistent with the HRM and GCP classification results obtained using F1R1.

### 3.4. Pilot Proof-of-Concept Application to Direct Milk Samples

To assess the applicability of the HRM workflow to clinical matrices, DNA extracted directly from selected clinical milk samples was analysed using the F1R1 primer pair. These samples represented *Escherichia coli*, *Streptococcus uberis*, *Staphylococcus aureus*, and *Staphylococcus chromogenes*. For each sample, the HRM curve generated from direct milk DNA was compared with the corresponding HRM profile obtained from the cultured isolate used as the species reference. In each case, the milk-derived HRM profile matched the corresponding cultured isolate profile, with comparable melting temperatures, curve shapes, and species assignments (Table 4). These findings provide a pilot proof of concept, based on four single-pathogen samples, that the HRM-based workflow can be applied directly to milk samples without prior culture under the conditions tested. In a clinical sample containing more than one pathogen, the resulting HRM melting profile would represent a composite of the individual species curves, which could alter peak shape and prevent reliable classification under the current single genotype GCP model; this scenario was not tested here and is discussed further in the Section 4 as a limitation.

## 4. Discussion

This study compared six 16S rRNA target regions for PCR-HRM-based differentiation of bacteria associated with bovine mastitis. By combining HRM profiling with GCP analysis, discriminatory performance was assessed objectively rather than by visual inspection alone. The findings show that not all 16S rRNA regions contribute equally to HRM classification. The differences in HRM discrimination observed across the six primer pair combinations most likely reflect the sequence characteristics captured within each amplified 16S rRNA region. Variation in melting temperature range, curve shape, and peak complexity is influenced by factors such as GC content, nucleotide distribution, and the presence of one or more melting domains within the amplicon [17]. Regions that capture greater sequence variability are more likely to generate distinct melt profiles, whereas more conserved regions tend to produce clustered curves with reduced separation. A single, sharp melting transition generally produces cleaner HRM profiles and supports clearer discrimination, whereas more complex melting behaviour can reduce resolution when sequence differences are unevenly distributed across the amplicon [17,23]. These findings reinforce that HRM performance is strongly region-dependent and that primer selection remains a critical determinant of discriminatory success.

Beyond its analytical performance, an assay of this kind has direct relevance to clinical decision making. Rapid species-level differentiation using the F1R1-based PCR-HRM workflow could support faster and more targeted antimicrobial selection, reduce unnecessary or ineffective treatment, and inform herd-level management decisions such as segregation or culling of persistently infected animals, complementing the general case for rapid diagnostics already outlined in the Introduction.

A major strength of the study was the use of GCP analysis to support objective classification. Visual inspection of HRM curves can indicate whether profiles appear distinct or overlapping, but it does not provide a quantitative measure of confidence [22]. In contrast, the use of species-specific cut-off values allowed each classification decision to be assessed against a defined threshold derived from repeated HRM runs. This is particularly important when analysing conserved targets such as the 16S rRNA gene, where biologically related species may still produce partially similar melt behaviour [17]. In this context, the classification of borderline profiles as variation rather than forcing species assignment improves specificity and reduces false-positive interpretation. The GCP analysis also showed that discriminatory performance differed more clearly among primer pairs than was apparent from visual curve inspection alone.

The reproducibility of HRM profiles and GCP values across repeated runs further supports the analytical stability of the method. Although small shifts in melting temperature and peak height were observed between runs performed at different times, the overall curve shape remained consistent for each reference genotype. Because the species-specific cut-off values were derived from repeated measurements of each reference genotype, their consistency reflects both inter-run reproducibility and the stability of the underlying HRM profiles. This is important for comparative analysis or diagnostic application, because minor fluctuations in temperature calling or fluorescence intensity should not substantially alter classification outcomes. Overall, these findings support the reliability of cut-off-based interpretation as part of a diagnostic workflow.

Comparative analysis across the six primer pair combinations confirmed that the targeted 16S rRNA region strongly influenced classification performance. Among the evaluated targets, F1R1 showed the most robust overall performance, whereas the remaining primer pairs showed varying degrees of cross-classification and reduced specificity. These findings indicate that a broader melting temperature range does not necessarily lead to the most reliable classification outcome. Rather, the most useful primer pair is the one that combines reliable amplification, stable melt behaviour, and clear separation from non-target species under the applied GCP model. This is consistent with previous HRM studies showing that target choice strongly affects discriminatory performance [29].

Sequencing of F1R1 amplicons provided independent confirmation that the classified isolates matched the expected species by BLAST analysis. The multiple sequence alignment showed nucleotide variation across the V1–V2 region and part of V3 that was consistent with the observed HRM differences. The phylogenetic tree derived from these sequences showed clustering broadly consistent with established taxonomic relationships, including broad separation between many Gram-positive and Gram-negative taxa. This region showed the best discriminatory performance, likely due to its higher sequence variability, short amplicon length (203 bp), interspecies differences in GC content, and favourable melting-domain characteristics that enhance HRM sensitivity to sequence differences. On the other hand, the shorter amplicon showed better HRM discrimination because it is more sensitive to small sequence differences. Longer amplicons may contain multiple melting domains that can mask or dilute the effect of local sequence variations, whereas shorter amplicons can produce more distinct and readily distinguishable melting profiles. However, this separation should be interpreted as phylogenetic clustering based on partial 16S rRNA sequence relatedness rather than direct phenotypic classification. Notably, phylogenetic divergence within an amplicon does not necessarily produce equally distinct melt patterns, because HRM profiles reflect the thermodynamic behaviour of the full fragment rather than phylogenetic relatedness alone [17,30,31].

HRM profiles generated directly from milk-derived DNA were consistent with those obtained from corresponding cultured isolates. This suggests that matrix-associated inhibitors did not substantially interfere with melt behaviour under the conditions tested. However, this application was limited to four selected samples and was intended as a proof of concept rather than a comprehensive clinical validation. Larger studies will be needed to determine sensitivity, specificity, and robustness across more diverse samples and pathogen loads [32,33].

Several limitations should be acknowledged. First, HRM discrimination remains constrained by the intrinsic variability of the targeted 16S rRNA region, meaning that no single amplicon is likely to provide complete species resolution across all taxa [34,35]. This was evident in the variable classification performance observed among the six primer pairs. Second, although GCP analysis substantially strengthened interpretation, it does not remove the biological reality that closely related taxa may share similar sequence features within conserved loci [17]. A related limitation is that only a single representative isolate was tested per species; the 16S rRNA gene, despite its overall conservation, can show intraspecific variability in some species. For example, multiple distinct 16S rRNA genotypes have been reported within *Staphylococcus chromogenes*, *Staphylococcus simulans*, and *Staphylococcus xylosus*, and *Streptococcus uberis* is known to show substantial 16S sequence heterogeneity. The absence of cross-classification observed for F1R1 in this study therefore reflects performance against the single reference isolate tested per species and may not extend to genetically diverse field isolates. Nevertheless, the bacterial panel included multiple Staphylococcus and Streptococcus species, and F1R1 was still able to discriminate these closely related taxa within the conditions tested. Third, the phylogenetic analysis was based on partial 16S rRNA sequences generated from the selected F1R1 region, which supports broad taxonomic interpretation but does not necessarily provide the same resolving power as longer or alternative molecular targets for all closely related organisms [34]. Finally, the direct milk analysis remained a proof of concept rather than a complete clinical validation. Furthermore, only a single representative isolate per bacterial species was evaluated. Therefore, the findings demonstrated the differentiation of tested isolates rather than comprehensive validation of species-level identification. Hence, further studies including multiple isolates per species should be used to evaluate intraspecific variability and confirm the broader applicability of the PCR-HRM assay. Moreover, as bovine isolates of these two species were unavailable, goat-derived isolates were included because they are recognised mastitis pathogens. The potential influence of host-related strain variation is acknowledged as a study limitation. As individual animal-level identifiers were not available for the diagnostic submissions (Section 2.1), the panel cannot be confirmed to represent 547 independent animals, and the possibility that some samples originated from the same cow cannot be excluded; this should be considered when interpreting the diversity and representativeness of the isolate panel.

Overall, the findings indicate that PCR-HRM provides a rapid, cost-effective, and high-throughput approach for preliminary discrimination of mastitis-associated bacteria when supported by an objective GCP-based classification model. Within the current bacterial panel, F1R1 showed the most consistent overall performance for discrimination. Discrimination among 18 clinically relevant mastitis-associated bacterial species demonstrates the potential of the PCR-HRM assay to provide rapid species-level identification, thereby supporting evidence-based treatment decisions and pathogen-specific mastitis control programs. However, these findings support further validation of PCR-HRM as a complementary screening approach in bovine mastitis diagnostics. Sequencing confirmed that the observed HRM profile differences reflect underlying nucleotide variation, supporting future comparison of unknown samples against validated reference melt profiles without requiring routine sequencing. We acknowledge that DNA extracted directly from milk containing multiple bacterial species may produce multiple amplicons and complex or intermediate HRM profiles, potentially resulting in misclassification.

## Figures and Tables

**Table 1:** Primer pairs targeting different regions of the 16S rRNA gene used for PCR-HRM analysis.

Primer Pair	Primer	Sequence (5′–3′)	Amplicon Size (bp)	Variable Region Coverage
F1R1	F1	CAGACTCCTACGGGAGGCAGCAG	203	V1–V2 and part of V3
	R1	GTGCCAGCAGCCGCGGTAATACG		
F2R2	F2	GTGCCAGCAGCCGCGGTAATACG	293	V3 and part of V4
	R2	AACAGGATTAGATACCCTGGTAGTCCA		
F3R3	F3	AACAGGATTAGATACCCTGGTAGTCCA	323	V6–V7
	R3	ATGTTGGGTTAAGTCCCGCAACG		
F1R3	F1	CAGACTCCTACGGGAGGCAGCAG	779	V1–V6 and part of V7
	R3	ATGTTGGGTTAAGTCCCGCAACGAGCGCAACCC		
F1R2	F1	CAGACTCCTACGGGAGGCAGCAG	473	V1–V3 and part of V4
	R2	AACAGGATTAGATACCCTGGTAGTCCA		
F2R3	F2	GTGCCAGCAGCCGCGGTAATACG	599	V3 fully and V4
	R3	ATGTTGGGTTAAGTCCCGCAACGAGCGCAACCC		

**Table 2:** Mean melting temperature values and GenBank accession numbers for 18 bacterial species analysed using six 16S rRNA primer pair combinations.

Bacteria	Tm (Mean ± SD, °C)						Accession No.
	Primer Pairs						
	F1R1	F2R2	F3R3	F1R3	F1R2	F2R3	
*Escherichia coli*	88.25 ± 0.00	89.17 ± 0.22	88.54 ± 0.77	90.05 ± 0.49	89.38 ± 0.23	89.11 ± 0.17	PZ169401
*Streptococcus uberis*	86.78 ± 0.32	87.80 ± 0.27	89.47 ± 0.59	89.57 ± 0.08	87.95 ± 0.40	89.35 ± 0.39	PZ169402
*Staphylococcus aureus*	86.47 ± 0.25	87.90 ± 0.24	88.43 ± 0.25	89.61 ± 0.14	87.93 ± 0.28	88.53 ± 0.63	PZ169403
*Streptococcus agalactiae* †	87.16 ± 0.77	87.93 ± 0.28	89.23 ± 0.33	89.52 ± 0.17	88.09 ± 0.45	89.03 ± 0.43	PZ169410
*Corynebacterium bovis*	89.29 ± 1.30	88.74 ± 1.14	89.73 ± 1.13	90.13 ± 1.00	89.80 ± 0.95	89.75 ± 0.50	PZ169414
*Serratia marcescens*	88.60 ± 0.12	89.13 ± 0.25	88.99 ± 0.17	89.70 ± 0.18	89.66 ± 0.61	89.07 ± 0.13	PZ169421
*Trueperella pyogenes*	88.06 ± 0.30	89.30 ± 0.29	89.91 ± 0.85	89.67 ± 0.31	90.08 ± 0.23	89.95 ± 0.32	PZ169422
*Staphylococcus chromogenes*	87.21 ± 0.13	87.93 ± 0.21	88.10 ± 0.19	88.55 ± 0.17	88.82 ± 0.16	88.41 ± 0.51	PZ169423
*Mannheimia haemolytica*	87.22 ± 0.41	88.02 ± 0.25	88.82 ± 0.15	88.77 ± 0.52	88.92 ± 0.15	89.43 ± 0.47	PZ169425
*Mycoplasma bovis*	87.06 ± 1.58	86.40 ± 0.54	85.64 ± 0.43	86.61 ± 0.24	87.01 ± 0.24	86.65 ± 0.46	PZ169429
*Staphylococcus simulans*	87.35 ± 0.04	88.28 ± 0.44	88.22 ± 0.56	88.39 ± 0.21	88.62 ± 0.20	89.29 ± 0.58	PZ169432
*Staphylococcus hyicus*	88.82 ± 0.86	88.13 ± 0.19	88.10 ± 0.74	88.80 ± 0.23	88.93 ± 0.14	89.49 ± 0.28	PZ169433
*Providencia stuartii* †	88.88 ± 0.37	88.15 ± 0.26	88.01 ± 0.67	88.98 ± 0.21	89.09 ± 0.15	88.17 ± 0.97	PZ169439
*Pantoea agglomerans*	89.71 ± 0.55	89.11 ± 0.12	88.93 ± 0.51	89.64 ± 0.08	89.87 ± 0.12	90.15 ± 0.45	PZ169438
*Streptococcus dysgalactiae*	88.40 ± 0.24	88.94 ± 0.97	88.98 ± 0.23	89.16 ± 0.31	88.91 ± 0.15	89.25 ± 0.46	PZ169501
*Klebsiella pneumoniae*	89.66 ± 0.52	89.53 ± 0.32	88.76 ± 0.47	89.73 ± 0.22	90.25 ± 0.17	89.73 ± 0.54	PZ169504
*Pasteurella multocida*	88.32 ± 0.30	88.15 ± 0.37	88.72 ± 0.14	89.34 ± 0.26	88.14 ± 0.30	87.99 ± 0.75	PZ169505
*Pseudomonas aeruginosa*	87.66 ± 0.19	88.19 ± 0.18	89.30 ± 0.47	90.48 ± 1.65	88.07 ± 0.41	89.00 ± 0.61	PZ169507

† Isolate derived from caprine milk.

**Table 3:** Summary of discriminatory performance across the six 16S rRNA primer pair combinations used for PCR-HRM analysis.

Primer Pair	Amplicon Size (bp)	Mean Tm Range Across Species (°C)	Visual Species Discrimination	GCP Classification Performance	Cross-Classification Observed	Overall Assessment
F1R1	203	86.5–89.7	Strong	Correct for all species	None observed	Best overall performer
F2R2	293	86.4–89.5	Moderate	Multiple cross-classification events	Multiple cross-classification events	Lower performance
F3R3	323	85.6–89.9	Lower	Multiple cross-classification events	Multiple cross-classification events	Lower performance
F1R3	779	86.6–90.5	Intermediate	Limited cross-classification	Limited cross-classification	Moderate performance
F1R2	473	87.0–90.3	Weak to moderate	Limited cross-classification	Limited cross-classification	Moderate to lower performance
F2R3	599	86.7–90.2	Strong	Selected cross-classification	Selected cross-classification	Second best overall performer

**Table 4:** Proof-of-concept application of the F1R1 PCR-HRM assay directly to selected clinical milk samples.

Milk Sample	Species Identified by Culture and MALDI-TOF MS	HRM Result from Direct Milk DNA	Reference Cultured Isolate Used for Comparison	Concordance
Milk 1	*Escherichia coli*	*Escherichia coli*	Corresponding *Escherichia coli* isolate	Yes
Milk 2	*Streptococcus uberis*	*Streptococcus uberis*	Corresponding *Streptococcus uberis* isolate	Yes
Milk 3	*Staphylococcus aureus*	*Staphylococcus aureus*	Corresponding *Staphylococcus aureus* isolate	Yes
Milk 4	*Staphylococcus chromogenes*	*Staphylococcus chromogenes*	Corresponding *Staphylococcus chromogenes* isolate	Yes

## Data Availability

The data supporting the findings of this study are available from the corresponding author upon reasonable request.

## Supplementary Materials

The following supporting information can be downloaded at https://www.mdpi.com/article/10.3390/microorganisms14091945/s1: Figure S1: Discrimination gap by reference genotype across 18 bacterial species. The discrimination gap was calculated as the difference between the self-match GCP and the highest non-target GCP for each reference genotype. Larger gaps indicate stronger separation from the closest non-target species, whereas smaller gaps indicate reduced separation and greater similarity to at least one non-target species. Abbreviations: EC, *Escherichia coli*; SU, *Streptococcus uberis*; SA, *Staphylococcus aureus*; STA, *Streptococcus agalactiae*; CB, *Corynebacterium bovis*; SR, *Serratia marcescens*; TP, *Trueperella pyogenes*; SC, *Staphylococcus chromogenes*; MH, *Mannheimia haemolytica*; MB, *Mycoplasma bovis*; SS, *Staphylococcus simulans*; SH, *Staphylococcus hyicus*; PR, *Providencia stuartii*; PN, *Pantoea agglomerans*; SD, *Streptococcus dysgalactiae*; KP, *Klebsiella pneumoniae*; PM, *Pasteurella multocida*; and PS, *Pseudomonas aeruginosa*. Table S1: Isolate panel comprising 18 mastitis-associated bacterial species. Table S2: The full set of species-specific GCP cut-off values for each primer pair.
