# Supplementary material for: Comparative Evaluation of 16S rRNA Target Regions Using PCR High-Resolution Melt Curve Analysis for Differentiation of Bacteria Associated with Bovine Mastitis
Source: Microorganisms. 2026 Sep 2;14(9):1945. doi: 10.3390/microorganisms14091945 (PMC13609044; doi:10.3390/microorganisms14091945)
Supplement: Supplementary file 1 [file microorganisms-14-01945-s001.zip › Table S1.pdf]

Supplementary Table S1. Isolate panel comprising 18 mastitis associated bacterial species.

| **Isolate code** | **Host species** | **Confirmed bacterial species** |
|---|---|---|
| M23-06102/9 | Bovine | *Escherichia coli* |
| M23-05678/12 | Bovine | *Streptococcus uberis* |
| M23-02577/29 | Bovine | *Staphylococcus aureus* |
| M24-11356/1A | Caprine | *Streptococcus agalactiae* |
| M24-06389/7F | Bovine | *Corynebacterium bovis* |
| M24-14244/1A | Bovine | *Serratia marcescens* |
| M24-06389/6A | Bovine | *Trueperella pyogenes* |
| M24-18210/1A | Bovine | *Staphylococcus chromogenes* |
| M25-01223/1A | Bovine | *Mannheimia haemolytica* |
| M24-14111/3B | Bovine | *Mycoplasma bovis* |
| M21-04333/1A | Bovine | *Staphylococcus simulans* |
| M21-01305/1A | Bovine | *Staphylococcus hyicus* |
| M21-02481/3B | Caprine | *Providencia stuartii* |
| M22-00813/6H | Bovine | *Pantoea agglomerans* |
| M24-06389/6B | Bovine | *Streptococcus dysgalactiae* |
| M24-10514/1A | Bovine | *Klebsiella pneumoniae* |
| M22-02041/9F | Bovine | *Pasteurella multocida* |
| M24-04405/B2 | Bovine | *Pseudomonas aeruginosa* |
